# Idiopathic Left Ventricular Tachycardia in an 11-Year-Old Boy

**DOI:** 10.1016/j.jaccas.2026.108489

**Published:** 2026-05-22

**Authors:** Adam DeLong, Michael Flaherty, Duraisamy Balaguru

**Affiliations:** Department of Pediatrics, Massachusetts General Hospital for Children, Boston, Massachusetts, USA

**Keywords:** awareness, electrocardiogram, ventricular tachycardia

## Abstract

**Image:**

Electrocardiogram demonstrating idiopathic fascicular left ventricular tachycardia.

**Case Summary:**

An 11-year-old boy with a history of tuberous sclerosis–related intractable epilepsy exhibited an idiopathic ventricular tachycardia that was resistant to multiple antiarrhythmic treatments, but was responsive to verapamil. We discuss the recognition and treatment of verapamil-sensitive fascicular left ventricular tachycardia.

**Take-Home Messages:**

Idiopathic fascicular left ventricular tachycardia in pediatric patients is an uncommon but recognizable arrhythmia. Its management differs from that of more commonly occurring pediatric wide-complex arrhythmias in that it readily responds to verapamil.

## Graphical Abstract

**Figure:**
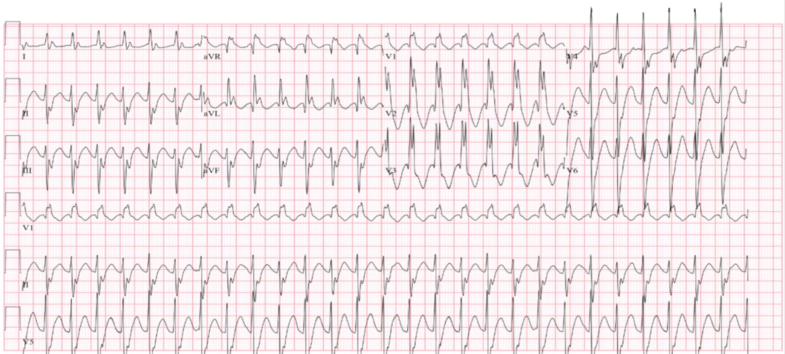

Idiopathic fascicular left ventricular tachycardia (IFLVT), also known as Belhassen ventricular tachycardia (VT) or verapamil-sensitive VT, is a reentrant-type tachycardia. IFLVT is readily recognizable on 12-lead electrocardiogram by its characteristic right bundle branch block pattern and left axis deviation, as well as by age range of occurrence, which notably includes pediatric patients.^1^^,^^2^ Therefore, pediatric physicians should be aware of this condition and its management.

An 11-year-old boy with a history of tuberous sclerosis–related intractable epilepsy was admitted for increased seizure frequency requiring benzodiazepine and barbiturate infusions, for which a percutaneously inserted central catheter (PICC) was placed. The patient was started on a norepinephrine infusion owing to sedation-related hypotension. During routine care, the patient was turned onto his side, and his rhythm changed from sinus tachycardia at 120 beats/min to a wide complex tachycardia at 176 beats/min (Figure 1). The patient was otherwise hemodynamically stable. Echocardiogram showed normal 4-chamber and valvar anatomy with a small, echogenic spot on the right side of the ventricular septum, speculated to be a remnant of a prior rhabdomyoma. A chest x-ray film demonstrated the PICC to terminate in the right atrium; the PICC was subsequently retracted to the cavoatrial junction. Intravenous lidocaine, esmolol infusion, procainamide, and 2 doses of adenosine (6 mg and 9 mg) were administered without any change in rhythm. Ultimately, 6 mg of verapamil administered intravenously over 5 minutes restored sinus rhythm.

**Figure 1:**
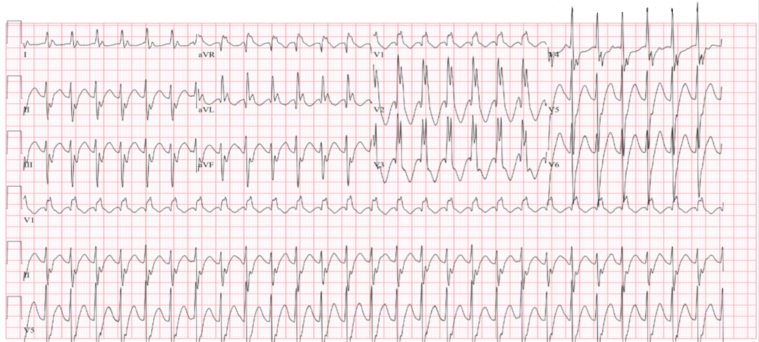
Patient Electrocardiogram Demonstrating Wide Complex Tachycardia 12-lead electrocardiogram shows wide complex tachycardia with right bundle branch block pattern and left axis deviation consistent with idiopathic fascicular left ventricular tachycardia in a 11-year-old boy with tuberous sclerosis and intractable seizures, which terminated with verapamil.

IFLVT is readily recognizable on a 12-lead electrocardiogram (Figure 1), exhibiting the following defining characteristics: monomorphic VT, right bundle branch block morphology, left axis deviation in the majority (90%) of cases, absence of structural heart disease (hence idiopathic), and sensitivity to verapamil.^1^^,^^2^ Causes of VT in children are provided in Supplemental Table 1.

Whereas most wide complex tachycardia in adults is VT, supraventricular tachycardia with aberrancy is more common in children.^3^ As such, IFLVT represents an important diagnosis for pediatric providers to recognize and treat correctly. Adenosine will abort most supraventricular tachycardia, but will not be effective for the majority of VT. Verapamil has been shown to be highly effective in aborting IFLVT, with synchronized direct current cardioversion being appropriate in unresponsive or unstable cases.^1^, ^2^, ^3^ Trial of other therapies as shown in the pediatric advanced life support (PALS) algorithm and used in the patient in this case can delay effective treatment for considerable duration.

The advanced cardiovascular life support (ACLS) algorithm for adults includes verapamil, but notably the PALS algorithm does not.^3^ Possible reasons for exclusion of verapamil from the PALS algorithm are relative contraindication in infants, whose myocytes are more sensitive to perturbations of extracellular calcium and calcium channels, and the relative risk of hypotension with verapamil use.^1^ Conversely, mentioning verapamil in the PALS algorithm or in the supporting materials could encourage pediatric physicians to consider verapamil earlier in appropriate cases of VT. This inclusion would also improve awareness of this entity among pediatric providers beyond pediatric electrophysiologists, including general pediatricians and pediatric physicians in emergency medicine, intensive care, and general pediatric cardiology.

## Funding Support and Author Disclosures

The authors have reported that they have no relationships relevant to the contents of this paper to disclose.
