# Supplementary material for: Idiopathic Left Ventricular Tachycardia in an 11-Year-Old Boy
Source: JACC Case Rep. 2026 May 22;31(26):108489. doi: 10.1016/j.jaccas.2026.108489 (PMC13326253; doi:10.1016/j.jaccas.2026.108489)

**Supplementary Table 1.** Causes of Ventricular Tachycardia in Children: Causes of ventricular tachycardia in children and adults are similar though the incidence of each cause may vary in different age groups.


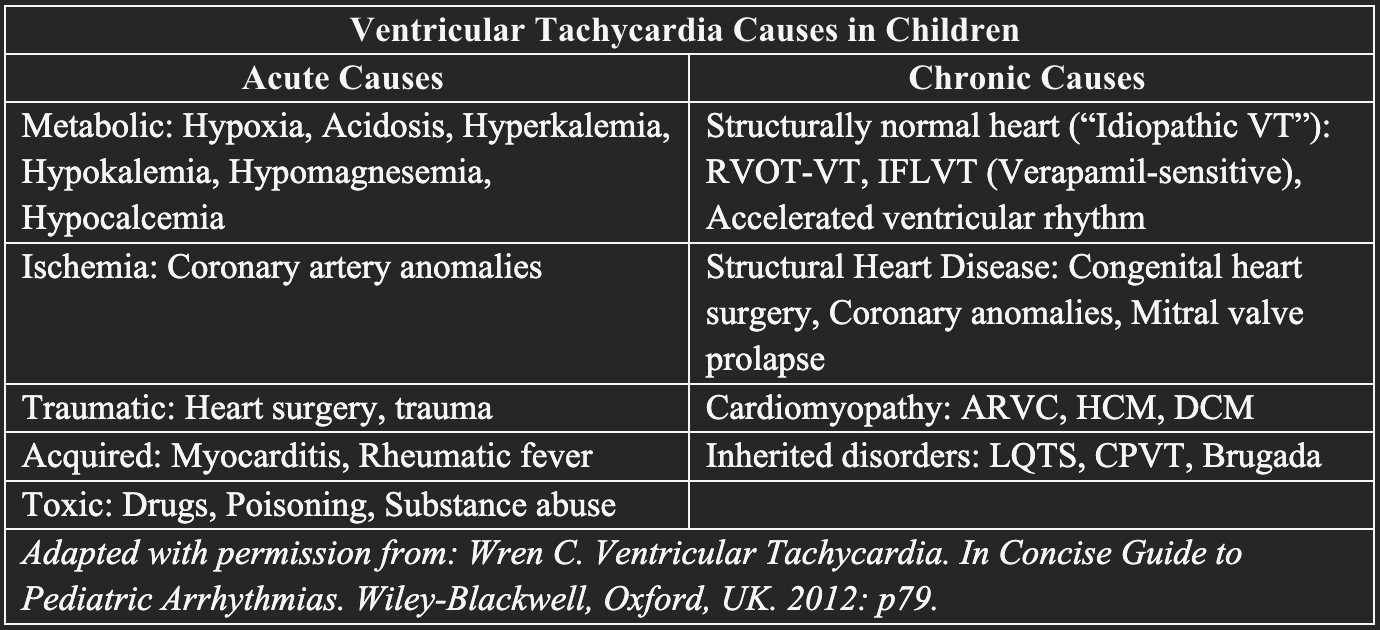

Supplement: Supplemental Table 1 [file mmc1.docx]
